# When Will Life Return to Normal? Parental Mental Health Post Quarantine and Extended Lockdown

**DOI:** 10.1192/j.eurpsy.2023.708

**Published:** 2023-07-19

**Authors:** D. Alonzo, M. Popescu, P. Zubaroglu

**Affiliations:** Fordham University, West Harrison, United States

## Abstract

**Introduction:**

Research exploring the mental health impact of the early stages of the pandemic among parents has found that 1 in 4 parents experienced increased psychological distress and reported more than one negative mental health symptom, especially quarantined parents. Ongoing stressors for parents related to the Covid-19 pandemic abound, yet the long-term impact of the pandemic on parental mental health remains largely unexplored, limiting the development and effectiveness of prevention and intervention efforts.

**Objectives:**

This study aims to fill this gap and explore the implications of the pandemic on parental mental health post-lockdown periods.

**Methods:**

The Covid Care Calls Program (CCC) was designed to address developing needs in response to the pandemic in highly marginalized communities across Guatemala. For the baseline study at the onset of the pandemic, 330 individuals participated. Convenience sampling was used. Callers administered a semi-structured interview to elicited information regarding health and mental health status, household composition, and nature of parenting and offspring functioning. For the current study, the same procedure of telephone surveying was used. A random sample of 100 baseline participants was included. Calls were administered between June 2021 and August 2021, 6-months post lifting of lockdown restrictions. Paired t-tests were used to examine differences in mental health impairment (anxiety, depression, stress, burnout) from immediately post-lifting of lockdown restrictions to 6-months following lifting of lockdown measures. Multiple linear regressions were used to examine predictors of mental health impairment.

**Results:**

We found that 6-months post lifting of stringent mitigation measures, parents reported engaging in increased negative parenting behaviors; observed increased negative behaviors from their children; reported increased anxiety, depression, and stress; and, reported increased burnout, especially mothers.

**Conclusions:**

Our findings regarding increased parental stress and burnout well after quarantine and lockdown periods are cause for concern. In addition to their own stress, children are likely to experience a negative downstream effect from their parents’ mental health impairment. Increased negative responses from children further exacerbate parental maladjustment which in turn increases negative child behaviors, and thus the cycle begins, resulting in an often conflictual, harsh, or disengaged home environment and dysfunctional parent-child relationships. This type of parental strain (has been noted to confer risk of psychopathology across generation. Family level intervention and increased access to community-based supports for parents are key to mitigating this persistent impairment.

**Disclosure of Interest:**

None Declared

